# Non-sedative cortical EEG signatures of allopregnanolone and functional comparators

**DOI:** 10.1038/s41386-022-01450-x

**Published:** 2022-09-27

**Authors:** Peter M. Lambert, Richard Ni, Ann Benz, Nicholas R. Rensing, Michael Wong, Charles F. Zorumski, Steven Mennerick

**Affiliations:** 1 Department of Psychiatry, Washington University in St. Louis School of Medicine, 660S. Euclid Ave., MSC 8134-0181-0G, St. Louis, MO 63110 USA; 2 Medical Scientist Training Program, Washington University in St. Louis School of Medicine, 660S. Euclid Ave., MSC 8134-0181-0G, St. Louis, MO 63110 USA; 3 Department of Neurology, Washington University in St. Louis School of Medicine, 660S. Euclid Ave., MSC 8134-0181-0G, St. Louis, MO 63110 USA; 4 Taylor Family Institute for Innovative Psychiatric Research, Washington University in St. Louis School of Medicine, 660S. Euclid Ave., MSC 8134-0181-0G, St. Louis, MO 63110 USA

**Keywords:** Ion channels in the nervous system, Pharmacology

## Abstract

Neurosteroids that positively modulate GABA_A_ receptors are among a growing list of rapidly acting antidepressants, including ketamine and psychedelics. To develop increasingly specific treatments with fewer side effects, we explored the possibility of EEG signatures in mice, which could serve as a cross-species screening tool. There are few studies of the impact of non-sedative doses of rapid antidepressants on EEG in either rodents or humans. Here we hypothesize that EEG features may separate a rapid antidepressant neurosteroid, allopregnanolone, from other GABA_A_ positive modulators, pentobarbital and diazepam. Further, we compared the actions GABA modulators with those of ketamine, an NMDA antagonist and prototype rapid antidepressant. We examined EEG spectra during active exploration at two cortical locations and examined cross-regional and cross-frequency interactions. We found that at comparable doses, the effects of allopregnanolone, despite purported selectivity for certain GABA_A_R subtypes, was indistinguishable from pentobarbital during active waking exploration. The actions of diazepam had recognizable common features with allopregnanolone and pentobarbital but was also distinct, consistent with subunit selectivity of benzodiazepines. Finally, ketamine exhibited no distinguishing overlap with allopregnanolone in the parameters examined. Our results suggest that rapid antidepressants with different molecular substrates may remain separated at the level of large-scale ensemble activity, but the studies leave open the possibility of commonalities in more discrete circuits and/or in the context of a dysfunctional brain.

## Introduction

Recently the FDA approved brexanolone, a formulation of the endogenous neurosteroid allopregnanolone (AlloP), as a rapid-acting antidepressant therapy for postpartum depression. Synthetic AlloP analogs may be useful for major depressive disorder [1, 2]. AlloP thus joins ketamine and possibly psychedelics as rapidly acting antidepressants [3–6]. Understanding features common among rapid antidepressants is important for more effective and selective neuropsychiatric treatments.

AlloP is a potent positive allosteric modulator (PAM) of GABA_A_ receptors (GABA_A_R), the primary fast inhibitory neurotransmitter receptor in the CNS [7]. GABA_A_Rs are heteropentameric ligand gated chloride channels found in virtually all CNS neurons. Although nineteen GABA_A_R subunits have been identified, most functional native receptors are composed of two α, two β, and a variable fifth subunit. The identity of these subunits confers physiologic and pharmacologic receptor properties. For example, receptors containing a γ2 subunit are typically synaptic and drive phasic inhibition, while receptors containing a δ or α5 subunit are typically extrasynaptic and mediate tonic inhibition.

Subunit selectivity is believed to underlie actions of some clinically important drugs, including benzodiazepines and neurosteroids. Benzodiazepines require certain α subunits and bind at the interface of α and γ subunits and thus lack activity at δ containing GABA_A_Rs [8]. Modulation at the benzodiazepine site can affect both phasic inhibition through α1/2/3 containing receptors, and tonic inhibition through actions at α5 containing GABA_A_Rs, which are located primarily on excitatory pyramidal cells [9, 10]. While benzodiazepines produce reliable anxiolytic effects, they lack clinically useful antidepressant activity. Neurosteroids, including AlloP, enhance both phasic and tonic inhibition and may act preferentially at δ-containing GABA_A_Rs [11–13]. Barbiturates are broad spectrum GABA_A_R PAMs [14, 15] but could share selectivity for δ-containing receptors with neurosteroids [16]. δ-containing receptors are commonly found on principal neurons where they are usually part of α4βδ or α6βδ pairings [17, 18]; however they are also expressed by some interneurons, particularly parvalbumin positive (PV + ) fast spiking interneurons, where they comprise a unique α1βδ pairing that has shown different pharmacologic properties from classic δ subunit pairings in vitro [19, 20].

To develop even more effective and selective treatments, it is necessary to expand our understanding of AlloP and its modulation of brain circuits and network activity. The coordinated activity of neurons gives rise to brain rhythms and extracellular field oscillations that can be measured on the cortical surface with electroencephalograms (EEG). The activity of interneurons is largely responsible for maintaining network oscillations. In particular, gamma oscillation frequency is dependent on the decay kinetics of inhibitory postsynaptic potentials on pyramidal cells produced by GABA release from PV + interneurons [21]. Additionally, the prolongation of these decay kinetics by diazepam can modulate the frequency of oscillatory rhythms in EEG signals including slowing theta and gamma oscillations in mice, and potentiating beta frequency oscillations in rodents and humans [22, 23]. Study of EEG alterations by AlloP can inform about neurosteroid neuromodulation integrated across diverse cell types over multiple circuits, presumably relevant to complex behaviors. EEG is also readily applied to both rodents and humans. Comparison of oscillatory modulation with that induced by other known rapid antidepressants may yield insight into shared network-level signatures of antidepressant drugs that could be used to screen for new antidepressant activity.

While novel rapid-acting antidepressants such as AlloP and ketamine produce persisting effects that outlive drug presence in the brain, antidepressant effects emerge rapidly. Therefore, study of network level activities acutely modulated by AlloP and other rapid acting antidepressants may reveal common changes among otherwise distinct drugs and help to inform the further development of new antidepressants. In addition, differentiating network modulation induced by AlloP from other drugs that share effects on GABA_A_Rs, including the selective actions of benzodiazepines described above, can help elucidate the mechanism through which AlloP induces its antidepressant response.

Here we utilized video-EEG recordings in freely behaving mice to characterize the network level effects of AlloP at a sub-sedative dose. We included two categories of comparator drug treatments. We first compared AlloP effects to those of pentobarbital and diazepam, two GABA_A_R PAMs that lack known antidepressant effects. Pentobarbital may lack the subunit-selective effects of AlloP and diazepam outlined above [14–16]. Second, we compared EEG effects of AlloP and another rapid acting antidepressant, ketamine, to identify shared alterations to network activity that may indicate a convergence of these two drugs with different molecular targets toward network effects. AlloP robustly increased spectral power in beta (12–30 Hz) and low gamma (30–55 Hz) frequency ranges during active wake, a feature shared with pentobarbital. Diazepam, which has more selective actions at GABA_A_R populations, had weaker effects on this frequency range than AlloP or pentobarbital. There were few similarities between the acute effects of AlloP and ketamine on EEG. Similar comparative patterns characterized changes to functional connectivity and cross-frequency coupling by the drugs. Overall, the results indicate that ketamine and AlloP differentially modulate network activity during the early phase of drug action. Our results raise questions about the antidepressant efficacy of barbiturates and the selectivity of AlloP.

## Methods

### Drugs

AlloP (Sigma) was initially dissolved in 45% 2-hydroxypropyl β-cyclodextrin (CDX) at a concentration of 1.2 mg/mL and sonicated until completely dissolved, then further diluted in sterile saline (0.9% NaCl) to 0.6 mg/mL AlloP and 22.5% CDX. Pentobarbital (Sigma) and ketamine (Sigma) were both dissolved in sterile saline to final concentrations of 6 mg/mL and 2 mg/mL respectively. Diazepam (Sigma) was dissolved in 40% propylene glycol in sterile saline at a concentration of 0.2 mg/mL. All drugs were delivered as a single intraperitoneal injection with the following doses (mg/kg): AlloP 5, pentobarbital 15, diazepam 1, ketamine 10. Dosing was determined by pilot studies ensuring no loss of righting reflex in the hour following injection to target the sub-anesthetic dose range. AlloP and ketamine doses were within ranges previously shown to produce antidepressant-like effects in rodents [24–27].

### EEG surgery

Mice (C57BL6/J JAX# 000664) of both sexes were anesthetized with isoflurane (5% for induction, 1.5–2% for surgery), and mounted in a stereotactic frame (Kopf, Tujunga, CA). Bilateral holes were drilled in the skull for insertion of epidural screw electrodes for frontal (+0.7 AP, ± 0.5 ML bregma), and parietal (-2.0 AP, ± 1.5 ML bregma) electrodes. An additional screw over the cerebellum (-1.0 AP lambda) served as a common ground reference. To facilitate vigilance scoring of EEG, a single stainless steel wire was implanted in the nuchal muscle for EMG measurement. Animals were allowed to recover in their home cages for three days before initiating EEG recordings.

### EEG recording

For the duration of the experiment mice were maintained on reverse lighting cycle and recordings were initiated in the first half of the dark cycle to enrich for active wake behaviors throughout the period of acute drug exposure. EEG was acquired from four mice simultaneously with each recording chamber containing a 16ch RHD headstage with 3-axis accelerometer (Intan technologies, C3335) controlled by a single OpenEphys acquisition board via the OpenEphys GUI. Signals were digitized at 1000 Hz and filtered from 0.1 to 250 Hz with a 2nd-order Butterworth digital filter. A series of 5 cohorts of 4 animals each were recorded for a total of 5 animals per drug group (AlloP 3 M/2 F, pentobarbital 5 M/2 F, diazepam 3 M/2 F, ketamine 3 F/2 M). Animals were briefly habituated to tethering and the recording chamber for 2 h at least one day prior to the experimental session. Recordings for the experimental session began with a 30 min baseline recording period. Next a vehicle injection for each drug condition was delivered, followed 30 min later by the active drug. EEG monitoring continued for 12 h.

### EEG analysis

Raw data were imported into MATLAB for further analysis. Time frequency spectrograms were generated from a wavelet transform of the raw EEG signal, utilizing a set of 100 complex morlet wavelets centered from 1 to 100 Hz in 1 Hz steps with wavelet cycles increasing logarithmically from 3 to 30. 5 min of artifact-free EEG in which active wake was the dominant behavioral state were identified from the baseline, vehicle, and drug periods. Segments were identified by combined evidence of animal movement from video, EMG, and accelerometer, and the presence of theta rhythm in the parietal electrode. Typical behaviors defining these segments included digging, ambulation, and rearing. Oscillations were detected using the Better OSCillation (BOSC) method [28] which incorporates both power and duration thresholds to detect true oscillations, and produces the measure P_episode_, representing the proportion of the time segment analyzed that an oscillation at a given frequency was present. Raw power spectra calculated with traditional FFT based methods confirmed main drug effects observed with the BOSC method (Supplementary Methods, Fig. S1). Coherence was calculated between ipsilateral frontal and parietal electrodes over 5 min of active wake EEG using the multitaper coherency method in the Chronux MATLAB toolbox [29, 30]. The coherencenysegc function was used with window length of 5 s and taper parameters [TW, K] = 7.5, 14 to calculate coherency of oscillations <100 Hz. Coherency results were collapsed further into bins of 5 consecutive estimates before statistical testing. Theta-gamma phase amplitude coupling was computed during the same segments of active wake using the modulation index measure [31]. Briefly, low frequency phase was extracted from the Hilbert transform of a series of bandpass filtered signals centered from 4 to 15 Hz with 2 Hz bandwidth in 1 Hz steps, and high frequency amplitude was extracted from the Hilbert transform of a series of bandpass filtered signals centered from 15 to 100 Hz with 20 Hz bandwidth in 5 Hz steps. The binning of high frequency amplitudes by instantaneous low frequency phase allowed for the calculation of a modulation index for each combination of low and high frequencies.

### Statistics

For all drug conditions, statistical comparisons were made to the vehicle injection period. Frequency distributions of P_episode_ were assessed with a repeated measures two-way ANOVA with factors of drug treatment and oscillation frequency, followed by Dunnett’s multiple comparisons test for each frequency of oscillation. Frequency bands for bandpower calculations were defined as described previously [32], and the integral of the P_episode_ distribution within each frequency band was normalized to the vehicle condition to allow for direct assessment of drug effects on bandpower. A two-way repeated measures ANOVA was performed for each drug and electrode to assess drug effects on vehicle-normalized bandpower compared to the respective baseline conditions, followed by Dunnett’s multiple comparisons test for each frequency band. Comparison of other drugs to the effects of AlloP was assessed for vehicle normalized P_episode_ distributions and bandpower with a standard two-way ANOVA followed by Dunnett’s multiple comparisons test comparing effects of each drug to AlloP. Coherence was tested with a two-way repeated measures ANOVA, followed by Dunnett’s multiple comparisons test for each frequency bin. A threshold of 5 continuous frequency bins (2.44 Hz bandwidth) was considered a meaningful difference for multiple comparisons. Comparisons of summary statistics are presented as mean ± SEM along with an estimate of the group differences ± 95% confidence interval calculated from the Dunnett’s multiple comparisons test.

All results reported here, excluding coherence measures, were part of a ‘hypothesis testing’ phase of experiments, preceded by similar pilot studies (hypothesis generating) performed on a smaller group of animals in a drug crossover design (*n* = 4 mice treated with AlloP and ketamine). Additional pilot experiments titrated dosages to ensure just sub-sedative effects. Analysis approaches were worked out during the hypothesis generating phase, and these preliminary results showed evidence for depressed theta-gamma coupling for ketamine and AlloP, a commonality between antidepressant drugs that did not replicate in the hypothesis testing phase. Coherence measures were added during the later phase and thus can be considered hypothesis-generating.

## Results

### Acute effects of sub-sedative AlloP and comparators on cortical EEG

**Fig. 1:**
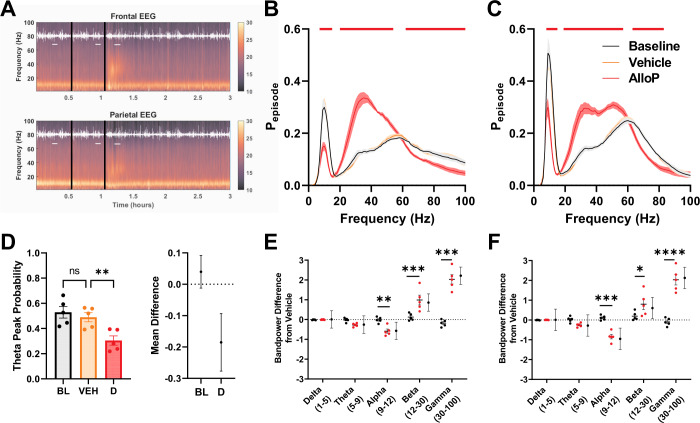
Sub-sedative AlloP acutely alters mid frequency range oscillations in active wake EEG. **A** Representative spectrograms showing time course of acute drug exposure including baseline, vehicle, and drug periods. Black lines indicate vehicle and drug injection, horizontal white lines indicate active segments used for further analysis, and white overlay represents animal activity measured via accelerometer. **B**, **C** AlloP effects on frontal (**B**) and parietal (**C**) active wake EEG oscillations, represented as the proportion of analyzed time segment that an oscillation at a given frequency was present (P_episode_; see Methods). Repeated measures two-way ANOVA showed an interaction between drug treatment X oscillation frequency at both electrodes analyzed (Frontal – F(198,800) = 41.14, *p* < 0.0001, Parietal – F(198,800) = 33.59, *p* < 0.0001). Horizontal bars indicate difference from vehicle after Dunnett’s multiple comparisons testing (black – baseline, orange – vehicle, red – AlloP (5 mg/kg)). **D** Peak P_episode_ of theta oscillations in parietal EEG. Right panel shows 95% confidence interval of mean difference of P_episode_ estimate. One-way repeated measures ANOVA revealed an effect of treatment on P_episode_ (F(1.498, 5.991) = 49.38 *p* = 0.0003). Dunnet’s multiple comparison showed a difference between Vehicle (VEH) and AlloP (Drug, **D**) peak (*p* = 0.0045). Baseline (no injection) is denoted as BL. **E** Integrated bandpower of vehicle-normalized P_episode_ distribution from frontal electrode. Dotted line indicates vehicle (normalizing condition). Black symbols denote the baseline condition. Red symbols denote AlloP. Two-way repeated measures ANOVA showed drug treatment X frequency band interaction (F(4, 20) = 52.20 *p* < 0.0001). Dunnett’s multiple comparisons showed difference between vehicle and AlloP for alpha (*p* = 0.0075), beta (*p* < 0.0001) and gamma (*p* < 0.0001) band power. **F** Integrated band power of vehicle-normalized P_episode_ distribution from parietal electrode. Two-way repeated measures ANOVA revealed drug treatment X frequency band interaction (F(4, 20) = 37.41 *p* < 0.0001). Dunnett’s multiple comparisons showed difference between baseline and AlloP for alpha (*p* = 0.0003), beta (*p* = 0.0241) and gamma (*p* < 0.0001) band power. Black circles and bars to the right of treatment groups in (**E**, **F**) represent estimate of mean difference between drug and baseline treatment ± 95% confidence intervals calculated from Dunnett’s multiple comparisons test.

To standardize the effect of behavioral state on EEG signals, we focused on EEG changes in the active wake behavioral state during the period of acute drug action. The dominant features of the baseline active wake EEG are a robust theta frequency oscillation, especially prominent in parietal electrodes, and the presence of oscillations in the broad gamma frequency range. Injection of CDX vehicle failed to alter either of these EEG features. A single intraperitoneal injection of 5 mg/kg AlloP showed rapid onset of EEG changes during active wake, with effects persisting for up to 45 min (Fig. 1A). The most prominent feature induced by AlloP was an increase in beta (12–30 Hz) and low gamma (30–55 Hz) frequency oscillations detected in all measured electrodes (Fig. 1B, C). Notably, this increase in mid frequency range oscillations was more prominent in frontal electrodes, which have lower baseline power at these frequencies (Fig. 1B), than the parietal electrodes (Fig. 1C). Additionally, AlloP reduced the level of high gamma frequency oscillations. As expected from active wake, parietal electrodes showed prominent theta oscillations, likely hippocampal driven, that were reduced by AlloP (Fig. 1D). When normalized to the vehicle period, AlloP increased EEG power in the beta and gamma bands compared to vehicle injection, coupled with a reduction in alpha band power (Fig. 1E, F). We did not observe evidence of a sex effect in the acute EEG response to AlloP during active wake (Fig. S2), although the study was not powered to detect small differences. Active wake segments sampled from hours 6–9 and 9–12 of the recording session confirmed a return to baseline EEG signatures (Fig. S3), indicating the above findings represented acute effects of AlloP on network activity.

**Fig. 2:**
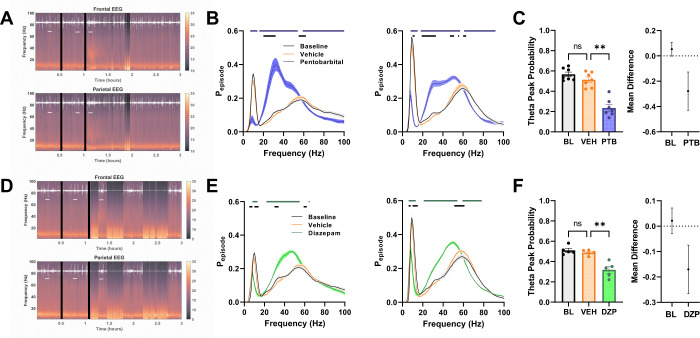
Pentobarbital and diazepam share features with AlloP on EEG. Representative spectrograms showing time course of acute effects of pentobarbital (**A**) and diazepam (**D**) on frontal (top panels) and parietal (bottom panels) EEG. Black lines indicate vehicle and drug injection, horizontal white lines indicate active segments used for further analysis, and white overlay is trace from head-mounted accelerometer. **B** Pentobarbital effects on frontal (left panel) and parietal (right panel) active wake EEG oscillations. Two-way ANOVA showed frequency X drug interaction in both frontal (F(198, 1200) = 39.29, *p* < 0.0001) and parietal (F(198, 1200) = 51.17, *p* < 0.0001) electrodes. **C** Parietal theta rhythm peak was reduced (one-way ANOVA, F(1.204, 7.227) = 32.89, *p* = 0.0001). Dunnett’s multiple comparisons test between vehicle and pentobarbital revealed a difference (*p* = 0.0005). **E** Diazepam induced changes in frontal (left panel) and parietal (right panel) active wake EEG oscillations. Two-way ANOVA showed frequency X drug interaction for frontal (F(198, 800) = 30.72, *p* < 0.0001) and parietal (F(198, 800) = 31.48, *p* < 0.0001) electrodes. **F** Theta frequency peak was reduced during acute diazepam effects (One-way ANOVA, effect of drug treatment on peak F(1.218, 4.870) = 47.24, *p* = 0.0009). Dunnett’s multiple comparisons showed difference between vehicle and diazepam (*p* = 0.0072). Horizontal lines in **B**, **E** represent difference from vehicle spectra following Dunnett’s multiple comparisons test with an alpha threshold of 0.05.

To investigate how the effects of AlloP compared with behaviorally similar doses of other GABA_A_R PAMs, we compared AlloP to pentobarbital and diazepam, two GABA_A_R PAMs with different degrees of subunit selectivity. We found that both pentobarbital and diazepam altered EEG with a timescale similar to that of AlloP (Fig. 2A, D). Although we selected a dose of diazepam that preserved enough active wake behavior necessary for our intended analysis during acute drug exposure, the animals receiving diazepam did exhibit more sleep behavior during the hours immediately following drug injection (Fig. S4). Similar to the oscillatory changes induced by AlloP, the acute effects of pentobarbital and diazepam were strongest in mid frequency range oscillations (Fig. 2B, E). Interestingly, pentobarbital induced oscillations lower in the beta range (peak at ~35 Hz) than the oscillations induced by diazepam (peak at ~50 Hz), and pentobarbital increased the mid frequency oscillations more than diazepam. Additionally, both GABA_A_R PAMs decreased the strength of the theta rhythm (Fig. 2C, F), similar to AlloP. Overall, pentobarbital recapitulated all the changes evident with AlloP, while diazepam only partially recapitulated the effects of the other GABA PAMs.

**Fig. 3:**
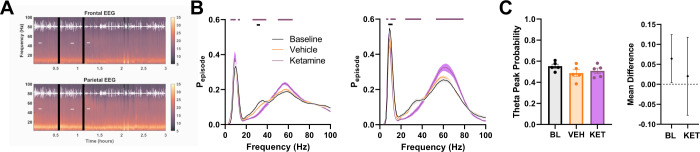
Ketamine EEG effects segregate from those of AlloP and GABA_A_R PAMs. **A** Representative spectrogram showing time course of decrease in beta in frontal EEG (top) and increase in high-range gamma power in parietal EEG (bottom) following ketamine injection, black lines represent vehicle and drug injection, horizontal white lines indicate active segments used for further analysis, white overlay is motion detected from accelerometer. **B** Ketamine effects on frontal (left) and parietal (right) EEG oscillations during active wake. There were decreases in beta frequency range and increase in mid-gamma power. Two-way repeated measures ANOVA showed drug X frequency interaction for frontal (F(198, 800) = 7.743, *p* < 0.0001) and parietal (F(198, 800) = 13.63, *p* < 0.0001) EEG. Horizontal lines indicate difference from vehicle spectra based on Dunnett’s multiple comparisons. **C** Theta frequency peak in parietal EEG did not differ between vehicle and drug treatments. One-way ANOVA failed to detect main effect of drug treatment (F(1.538,6.153) = 2.990, *p* = 0.1285).

In efforts to find common patterns of altered network activity induced by rapid-acting antidepressants, we compared the effects of AlloP to those induced by a sub-sedative dose of ketamine, an NMDA receptor antagonist that exhibits rapid antidepressant effects [25]. Following injection of ketamine, all mice remained active and showed drug-induced changes to the spectral content of both frontal and parietal EEG with rapid onset (Fig. 3A). Ketamine increased gamma frequency oscillations present during active wake, particularly in a higher end of the gamma range than the GABA_A_R targeting compounds (Fig. 3B). Moreover, unlike the compounds targeting GABA_A_Rs, ketamine decreased oscillations in the beta frequency range at both parietal and frontal sites (Fig. 3B, ~30 Hz). Interestingly, ketamine did not alter the strength of the theta frequency oscillations, based on one-way ANOVA (Fig. 3C), different from the other drugs in this study. However mean difference analysis suggested that vehicle lowered the theta peak probability (Fig. 3C, right). The basis of the apparent vehicle effect in Fig. 3C is unclear but could involve a small effect of acute stress, since pentobarbital and diazepam also showed small vehicle effects at limited frequencies within the theta band (Fig. 2B, E).

**Fig. 4:**
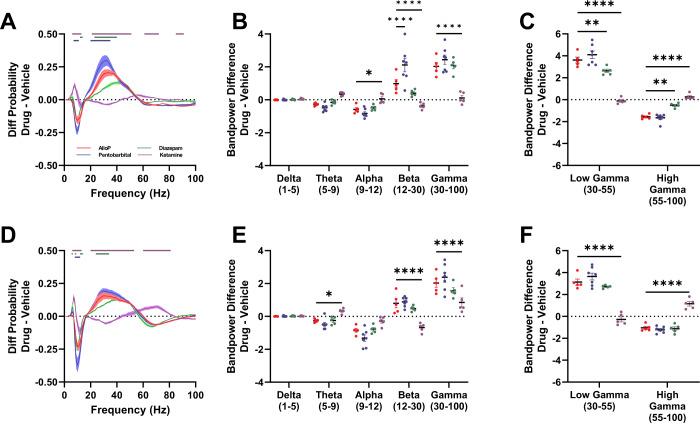
AlloP and comparators: Changes to active wake EEG. **A** Drug effects on frontal electrode active wake power spectra normalized to vehicle injection. Two-way ANOVA shows frequency X drug interaction (F(297,1800) = 17.75, *p* < 0.0001). **B** Drug-induced frontal EEG band power changes. Two-way ANOVA shows frequency band X drug interaction (F (12, 90) = 17.33, *p* < 0.0001). AlloP was treated as a standard to which other drugs were compared. AlloP differed from ketamine in alpha (*p* = 0.0405), beta (*p* < 0.0001) and gamma (*p* < 0.0001) bands. AlloP differed in beta from pentobarbital (*p* < 0.0001). **C** Drug-induced changes on gamma sub-bands of frontal EEG. Two-way ANOVA revealed frequency band X drug interaction (F (3,36) = 86.86 *p* < 0.0001). Dunnett’s multiple comparisons showed AlloP vs. ketamine (*p* < 0.0001) and AlloP vs. diazepam (*p* = 0.0078) differences in low gamma sub-band, and AlloP vs ketamine (*p* < 0.0001) and AlloP vs. diazepam (*p* = 0.0051). **D** Drug effects on parietal electrode active wake power spectra normalized to vehicle injection. Two-way ANOVA shows frequency X drug interaction (F(297,1800) = 16.97, *p* < 0.0001). **E** Drug-induced parietal EEG band power changes. Two-way ANOVA revealed frequency band X drug interaction (F (12,90) = 11.04 *p* < 0.0001). Dunnett’s multiple comparisons showed AlloP vs. ketamine difference in theta (*p* = 0.0486), beta (*p* < 0.0001), and gamma (*p* < 0.0001). **F** Drug-induced changes on gamma sub-bands of parietal EEG. Two-way ANOVA revealed frequency band X drug interaction (F (3,36) = 86.89, *p* < 0.0001). Dunnett’s multiple comparisons revealed AlloP vs. Ketamine difference in low (*p* < 0.0001) and high (*p* < 0.0001) gamma. Horizontal lines in **A**, **D** show significant difference compared to AlloP from Dunnett’s multiple comparisons test. AlloP – red, pentobarbital – blue, diazepam – green, ketamine – purple.

Similarities and differences among drugs are most easily visualized in side-by-side comparisons of spectral changes of all 4 drugs (Fig. 4). In Fig. 4, changes are expressed relative to respective vehicle, which showed relatively little difference from the uninjected state (baseline in Figs. 1–3, Fig. S5). Overall, the acute effects of AlloP on EEG oscillations are most similar to those of pentobarbital (Fig. 4A, D), a GABA_A_R PAM with broad subunit actions [14, 15]. Although diazepam also increased oscillations in the beta and low gamma ranges, it failed to reach the magnitude of the AlloP and pentobarbital effects (Fig. 4A, D). Interestingly, all four drugs in this study decreased parietal alpha band power (Fig. 4E). Ketamine had opposing effects to the GABA_A_R targeting compounds in the beta frequency range and was the only drug to potentiate oscillations in the high gamma sub-band in the parietal EEG (Fig. 4F).

### AlloP tracks with other GABA-A PAMs but not ketamine on measures of functional connectivity

**Fig. 5:**
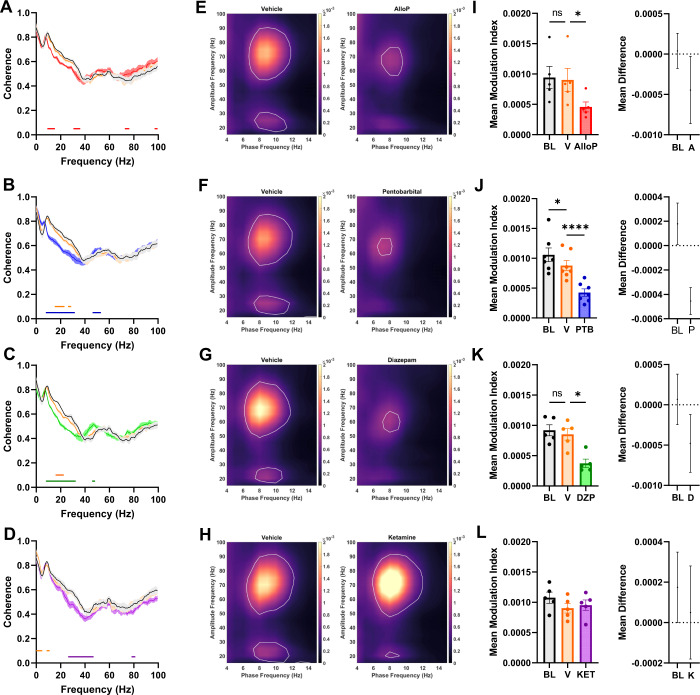
Coherence and phase amplitude coupling differentially altered by GABA_A_R PAMs compared to ketamine. **A**–**D** Coherence between ipsilateral frontal and parietal electrodes during baseline (black), vehicle (orange), and drug (**A** – AlloP (red), **B** – pentobarbital (blue), **C** – diazepam (green), **D** – ketamine (purple)). Two way repeated measures ANOVA revealed significant drug X frequency interaction for AlloP (F(326,1312) = 6.496, *p* < 0.0001), pentobarbital (F(326,1968) = 10.40, *p* < 0.0001), diazepam (F(326,1312) = 6.450, *p* < 0.0001), and ketamine (F (326,13120) = 2.385, *p* < 0.0001). Horizontal bars represent significant difference from vehicle condition from Dunnett’s multiple comparisons with alpha 0.05 and for > 5 continuous frequency bins. Theta-gamma comodulograms from 5 min of active wake during vehicle (left panels) and drug (right panels) periods. White contour line represents threshold of significant coupling strength after Bonferroni correction. AlloP (**E**, **I**), pentobarbital (**F**, **J**), and diazepam (**G**, **K**) all showed decreased coupling, with a shift to lower frequency pairs, while ketamine (**H**, **L**), did not acutely alter coupling. Right panels show effects baseline and drug relative to vehicle normalization, including confidence intervals. One-way ANOVA for modulation indices showed reduction by AlloP (F(1.435, 5.741) = 12.75, *p* = 0.0098), pentobarbital (F(1.334, 8.006) = 58.06, *p* < 0.0001), and diazepam (F(1.559, 6.235) = 12.66, *p* = 0.0078), with Dunnett’s multiple comparisons showing reductions between drugs and respective vehicle.

Coherence between oscillatory signals across brain areas represents a measure of functional connectivity [33], Here we used coherence between parietal and frontal EEG leads to assess functional connectivity changes induced during AlloP and comparator drug exposure. This coherence measure was previously employed to investigate the effects of ketamine on functional connectivity [34]. AlloP decreased the coherence between the frontal electrode and parietal electrode in the middle frequency range with reductions occurring around 15 and 35 Hz compared to the vehicle injection (Fig. 5A). These results suggest an acute reduction in functional connectivity between the frontal and parietal cortical areas during network modulation by AlloP.

To determine if the effects of AlloP on frontal-parietal EEG coherence are specific to neurosteroid action on GABA_A_Rs, we analyzed the effects of other GABA_A_R PAMs on coherence. Similar to our spectral analyses, AlloP affected coherence in a similar pattern to both pentobarbital and diazepam, which showed reductions in coherence throughout the beta frequency range (Fig. 5B, C).

In addition, we analyzed the effects of ketamine on coherence to investigate potential shared effects with AlloP (Fig. 5D). Compared to AlloP, ketamine injection primarily reduced frontal-parietal coherence in the lower end of the gamma frequency range (Fig. 5D), notably at frequencies higher than those that showed decreased coherence in the presence of AlloP.

### Decreased phase amplitude coupling during exposure to GABA_A_R PAMs but not ketamine

Among functional interactions in the brain, phase-amplitude coupling (PAC) of theta and gamma frequencies has received strong attention, as this measure is thought to be directly related to many cognitive behaviors that are disrupted in neuropsychiatric illness [35]. During active wake, the amplitude of higher frequency gamma oscillations is modulated by the phase of the slower theta rhythm [36]. We found that a single sub-sedative dose of AlloP reduced theta-gamma PAC (Fig. 5E, I). The decreased coupling was also observed in animals treated with pentobarbital (Fig. 5F, J) and diazepam (Fig. 5G, K). Similar to the pattern of results above, ketamine did not alter the strength of theta-gamma PAC, despite the overall increase in gamma frequency power (Fig. 5H, L). Taken together, the effects on theta-gamma PAC further suggest that AlloP modulates cross-frequency cortical network activity most similarly to a nonselective GABA_A_R PAM, with few similarities to ketamine, a drug that shares rapid antidepressant effects.

## Discussion

Here we compared in mice the effects of AlloP and several comparators on EEG signals during active exploration. All compounds were compared at a just sub-sedative dose during active wake. We also report the first assessment of theta-gamma cross-frequency coupling measured in EEG during AlloP and pentobarbital treatment. Both AlloP and pentobarbital promoted frontal oscillations at 15–55 Hz, including the beta and low gamma bands. AlloP and other GABA_A_R PAMs also disrupted higher order network organization measured by frontal-parietal coherence and theta-gamma PAC. Although we hypothesized that AlloP would distinguish from other non-antidepressant GABA_A_R PAMs, sub-sedative pentobarbital recapitulated with remarkable precision all AlloP-induced EEG signatures. Interestingly, diazepam, a more subunit-selective GABA_A_R PAM, showed a weaker increase of beta and low gamma frequency power. Finally, ketamine induced mostly distinct EEG features from the GABA PAMs. Several features (e.g., reduced parietal alpha bandpower) were shared by all drugs. Taken together, non-selective GABA_A_R PAMs, including AlloP, affect network activity very similarly, and ketamine’s actions are distinct, complicating efforts to identify a rapid antidepressant signature.

Previous studies have implicated increases in beta power as an EEG signature of GABA_A_R PAMs at non sedative doses [37]. Additionally, two neuroactive steroids with GABA_A_R PAM activity, alphaxalone and pregnanolone, increase beta frequency EEG amplitudes measured from 11.5 to 30 Hz during IV administration in rats while drug concentrations are in non-sedative ranges [38, 39]. More recently, zuranolone (SAGE-217), a synthetic AlloP analog in development for antidepressant treatment, increases beta power in EEG recordings from humans, rats, and mice [24, 40]. In our study, the increase in beta power during active wake was barely distinguishable from that of pentobarbital, suggesting that increased waking beta power is a reliable measure sensitive to sub-sedative broad-spectrum GABA_A_R PAM activity. While this signature is shared amongst GABA_A_R PAMs, increases in beta frequency power has been observed following sedative/hypnotic doses of non-GABAergic neurosteroids, as well as sedative doses of ketamine. However these increases in beta frequency power are accompanied by an increase in a broader low-frequency range power coupled with a shift out of active wake behavior [41–43]. The quantitative overlap between AlloP and pentobarbital was perhaps surprising given the purported selectivity of AlloP for δ subunit containing receptors mediating tonic inhibition [44–46]. Based on work in recombinant receptors, pentobarbital, like AlloP, increases agonist efficacy at low-efficacy δ-containing receptors [16]. However, pentobarbital sleep time is unaffected in δ null animals, different from neurosteroids [44]. Overall, our results suggest that at the sub-sedative dose used here, AlloP and pentobarbital behave similarly as nonselective GABA_A_R PAMs in vivo. We are tempted to speculate that barbiturates in fact may share desirable psychotropic properties with AlloP at sub-sedative doses if EEG signatures identify those desirable properties. Moreover, diazepam did not increase beta frequency power to the same degree as AlloP and pentobarbital, potentially due to its actions at GABA_A_R populations being limited to receptors containing a γ subunit [8]. Additionally, changes to network oscillations may depend on the cell types that are affected directly by each GABA_A_R PAM and the receptor subunit combinations present on their surface. For example, δ-containing receptors are found in α4βδ combinations on excitatory pyramidal cells and in α1βδ combinations on PV + interneurons [17–20]. The potential for AlloP and pentobarbital to modulate the α1βδ receptors primarily found on interneurons whose activity are essential for network oscillations further differentiates these two PAMs from diazepam. Regardless, the increase in beta and low gamma power continues to be a useful indication of network modulation through GABA_A_R activity since ketamine, an NMDA receptor antagonist, did not reliably increase EEG power in this range.

In addition to changes in average spectral power, study of the interaction between oscillations of different frequencies may aide in our understanding of how AlloP alters brain function. Coherence and cross frequency coupling between theta and gamma oscillations have been suggested to play an important role in cognitive processes [35, 47]. Alteration of theta-gamma coupling during acute drug exposure can assess the consequences of the drug effects propagated across the activities of many cell types that are crucial for maintaining these complex oscillatory interactions. Although we did not identify a difference between AlloP and other GABA_A_R PAMs, our results are consistent with those previously described for exposure to low dose diazepam [23] indicating that reduction in theta-gamma phase amplitude coupling likely characterizes all GABA_A_R PAMs. Interestingly, GABA_A_R PAMs commonly exert amnestic effects, which may reflect the ability of these drugs to disrupt higher order oscillatory dynamics relevant for cognitive functions such as theta-gamma coupling [48]. While previous reports have demonstrated that higher doses of ketamine increased theta-gamma coupling in hippocampal LFP recordings [49], we found that theta-gamma coupling was unaltered in the cortical EEG during acute exposure to the lower dose used here that is presumably relevant to antidepressant effects.

Despite the lack of unique signatures of AlloP effects on network activity during acute drug exposure, this finding doesn’t exclude acute or lasting commonalities between AlloP and ketamine, distinct from other GABA_A_R PAMs. Although our study represented a comprehensive evaluation of EEG oscillations, it is possible that local recordings (e.g., local field recordings) from relevant circuits would reveal commonalities among rapid antidepressants. For example, simultaneous recordings from ventral hippocampus and prefrontal cortex, a projection previously implicated in antidepressant effects of ketamine, may allow for a more nuanced comparison with AlloP [50]. Additionally, recordings from circuits relevant to behaviors associated with depressive-like phenotypes, such as reward circuitry relevant to anhedonia, may reveal common effects between rapid antidepressants that are not apparent in the cortical EEG signal [51, 52]. It is also possible that antidepressant drugs would exhibit similarities only in the context of dysfunctional brain activity. Indeed, our study can be considered a comprehensive baseline against which effects of drugs in various perturbed states can be compared. Further studies will be necessary to link direct actions of drugs on neural oscillations with potential corrections of dysfunctional baseline network activity. These studies will be a crucial aspect of identifying predictive EEG based biomarkers for antidepressant treatment response, which, to date, have failed to emerge reliably for more traditional antidepressant therapies [53].

Ketamine and AlloP antidepressant effects greatly outlive presence of drug. Thus, it is also possible that different triggers during acute drug action may lead to common persisting effects. Persisting effects of neurosteroids include changes in receptor composition [54, 55] or membrane trafficking of some GABA_A_R populations [56, 57]. Ketamine is not known to alter GABA_A_R receptor expression or trafficking but increases glutamate and BDNF signaling [58], which conceivably could lead to similar effects on circuits as AlloP effects.

It also remains possible that the AlloP exposure in our study was too brief to induce common changes to network activity. While a single injection of ketamine in mice or intranasal administration in humans can induce rapid and persistent antidepressant effects, the dosing protocol currently used in patients undergoing brexanolone treatment involves a considerably longer exposure before symptom improvements are observed relative to placebo treatment. Perhaps providing a longer duration of exposure to sub-sedative AlloP or multiple consecutive doses is necessary to produce circuit and network level responses different from those seen during the immediate acute exposure.

In summary, our work is the first to examine commonalities among anesthetics turned antidepressant: GABA_A_ PAMs and ketamine, with emphasis on mesoscale antidepressant triggers. At the level of cortical EEG, we find little evidence of commonalities between two distinct classes of rapidly acting antidepressants. Instead, we find remarkable similarity between the mesoscale actions of two broad-spectrum PAMs, despite differences in the purported receptor selectivity and clinical effects.

## Supplementary information

Supplemental Material
