# Supplementary material for: Non-sedative cortical EEG signatures of allopregnanolone and functional comparators
Source: Neuropsychopharmacology. 2022 Sep 27;48(2):371–9. doi: 10.1038/s41386-022-01450-x (PMC9751067; doi:10.1038/s41386-022-01450-x)
Supplement: Supplementary file 1 — Supplemental Material [file 41386_2022_1450_MOESM1_ESM.pdf]

Supplemental Methods

EEG Power Spectra Analysis

The 5 minutes of active wake for each electrode and condition were analyzed with the segmented continuous multi-taper FFT method from the Chronux toolbox in MATLAB. EEG data was segmented into 15 second windows and multi-taper parameters were [TW, K] = 15, 29. Power spectra were binned to a spectral resolution of 0.6 Hz before plotting.

Sleep Scoring

EEG/EMG was scored for sleep/wake stages with the AccuSleep toolbox for Matlab [1]. A subset of 15 second epochs were scored as Wake, NREM, or REM totaling a minimum of 5 minutes of each state. These were manually scored and used to calibrate a pretrained network, which subsequently scored the remainder of the epochs. A minimum of three successive epochs was required to classify a state change. Scored recordings were further reviewed by an expert scorer to validate proper scoring of state transitions. Data are shown as percent of each hour time bin spent in behavioral state.

Supplemental Reference

1. Barger Z, Frye CG, Liu D, Dan Y, Bouchard KE. Robust, automated sleep scoring by a compact neural network with distributional shift correction. PLoS One. 2019;14:e0224642.

Supplemental Figures

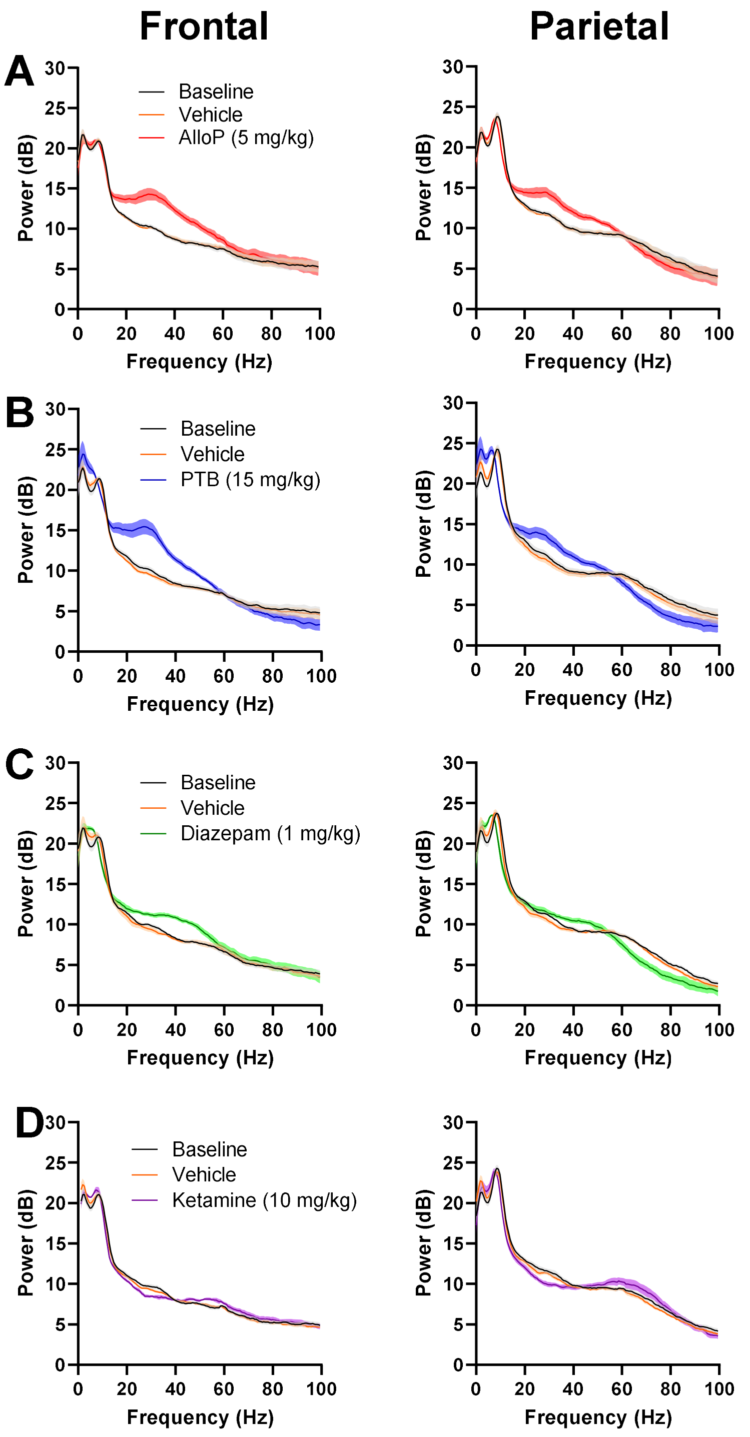

Supplemental Figure 1. Raw power spectra of drug responses in EEG data presented in Figs 1-3. (A – AlloP, B – Pentobarbital, C – Diazepam, D – Ketamine).

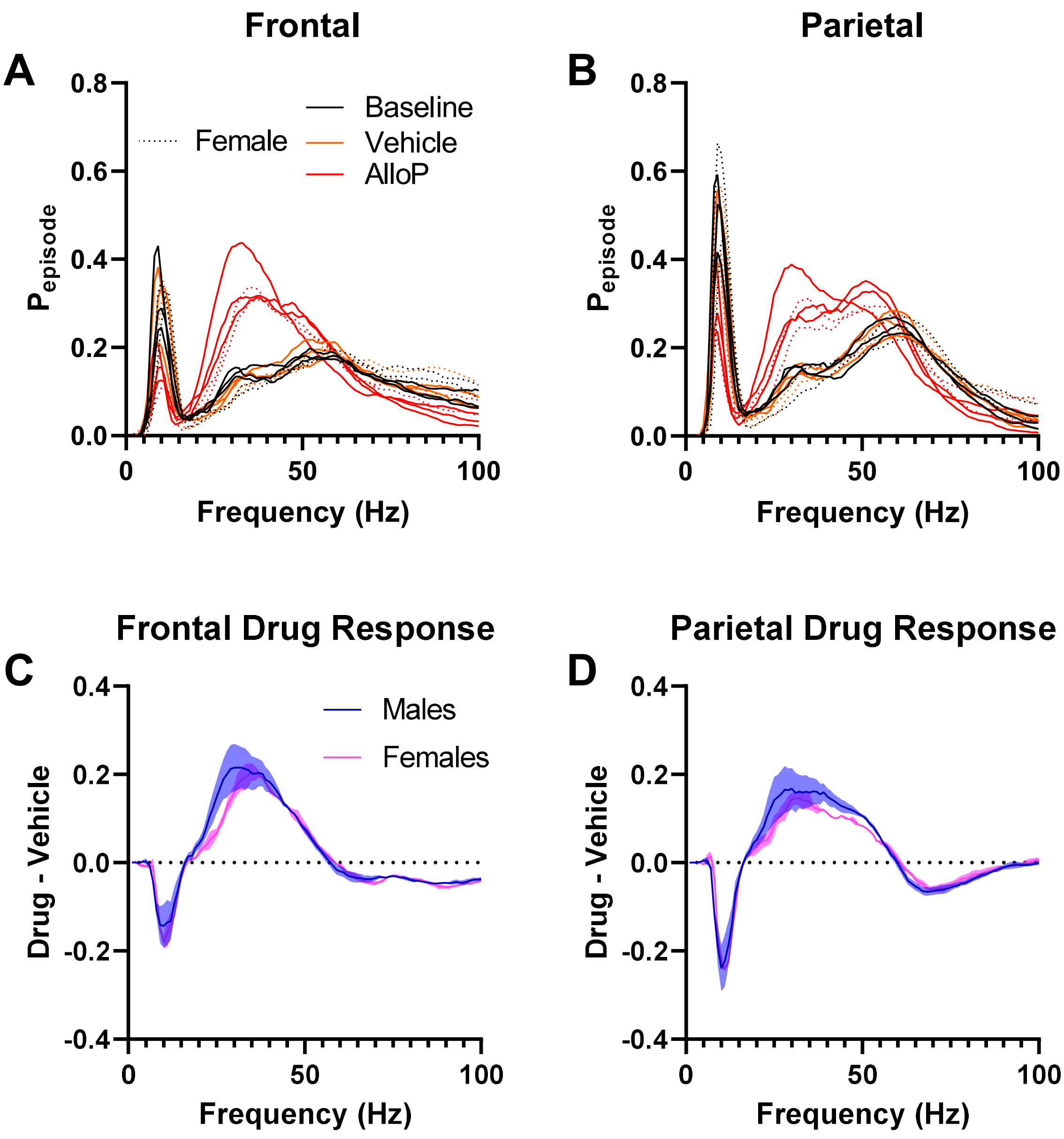

Supplemental Figure 2. AlloP responses by sex show similar drug induced changes for males and females during active wake. Individual animal responses plotted for (A) frontal and (B) parietal EEG oscillations (— Males, ····· Females). Vehicle normalized drug responses for (C) frontal and (D) parietal EEG showed almost overlapping frequency response during active wake segments.

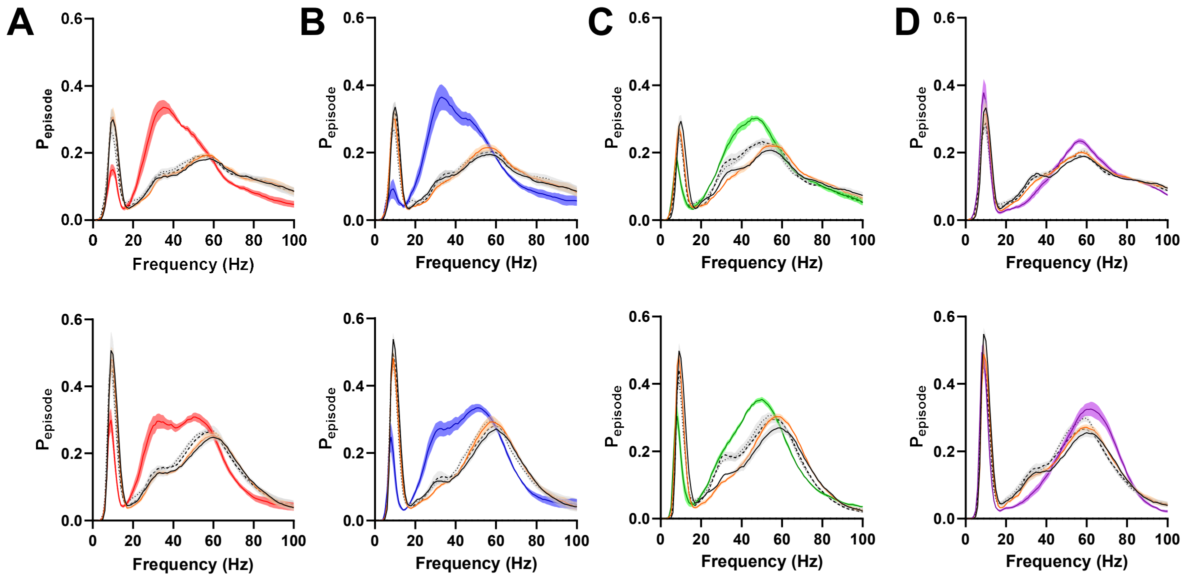

Supplemental Figure 3. Return to baseline EEG signatures following drug washout. Oscillations detected during active wake returned to baseline patterns for both frontal (top panels) and parietal (bottom panels) EEG in hours 5-9 (dashed lines) and 9-12 (dotted lines) of the recording. (A – AlloP, B – Pentobarbital, C – Diazepam, D – Ketamine).

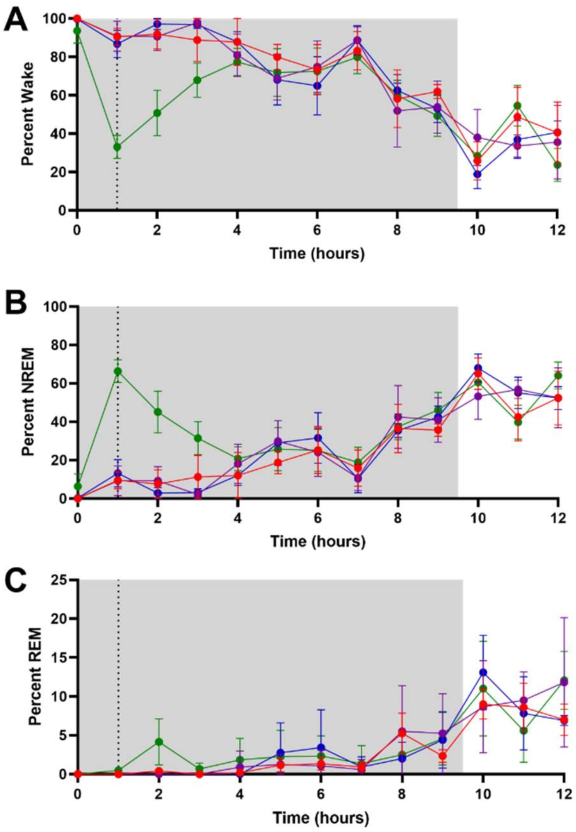

Supplemental Figure 4. Sleep and Wakefulness throughout EEG recording session Sleep staging for 1 hour time bins showing percent of each hour in (A) wakefulness, (B) NREM sleep, and (C) REM sleep. Dashed line indicates time of drug injection. Shading represents dark phase of light cycle. (AlloP – red, pentobarbital – blue, diazepam – green, ketamine – purple)

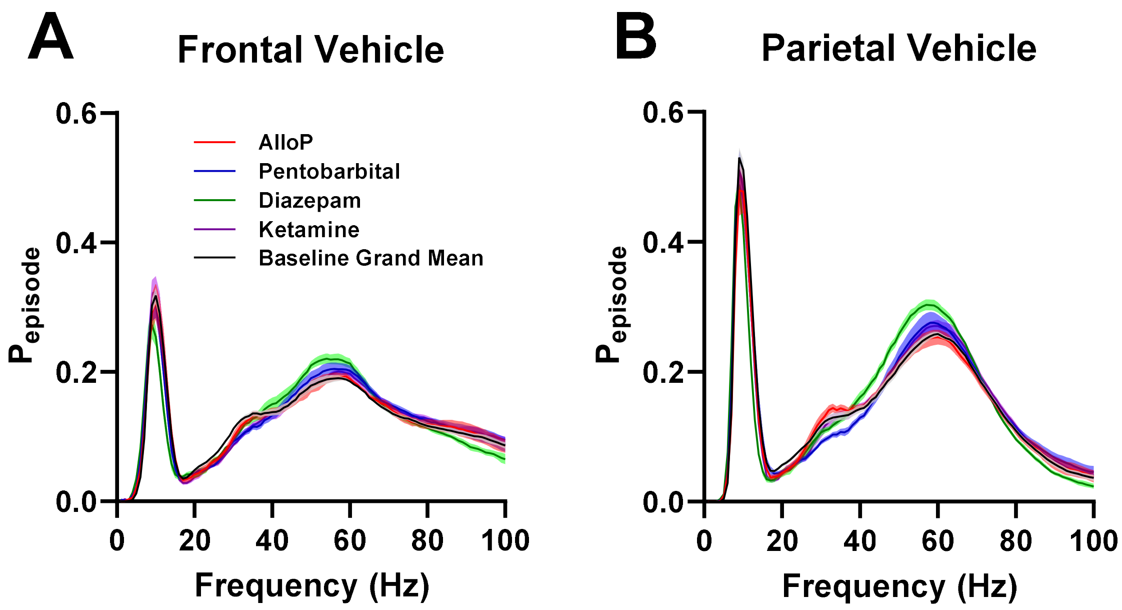

Supplemental Figure 5. EEG response to vehicle injection

Comparison of different vehicle effects on (A) frontal and (B) parietal EEG oscillations. (AlloP – 22.5% 2-hydroxypropyl β-cyclodextrin, Pentobarbital – 0.9% saline, Diazepam – 40% propylene glycol, Ketamine – 0.9% saline, Baseline – grand mean of uninjected state)
